# Idiopathic Ventricular Tachycardia in the Presence of Triple-Vessel Coronary Artery Disease: A Case Report

**DOI:** 10.7759/cureus.54955

**Published:** 2024-02-26

**Authors:** Nazima Khatun, Adam Kurnick, Jonathan Francois, Adam S Budzikowski, Louis Salciccioli, Sabu John

**Affiliations:** 1 Department of Internal Medicine, State University of New York Downstate Health Sciences University, Brooklyn, USA; 2 Department of Cardiovascular Medicine, State University of New York Downstate Health Sciences University, Brooklyn, USA; 3 Department of Cardiology, Kings County Hospital Center, Brooklyn, USA

**Keywords:** ventricular arrhythmia, outflow tract ventricular tachycardia, significant coronary artery disease, ventricular tachycardia (vt), idiopathic ventricular tachycardia

## Abstract

Exercise-induced ventricular tachycardia undergoes ischemia evaluation; however, it is important to identify idiopathic ventricular tachycardia in patients with concomitant coronary artery disease and radiofrequency ablations can be lifesaving. We report a case of exercise-induced right and left ventricular outflow tract ventricular tachycardia in a patient with triple vessel coronary artery disease.

## Introduction

Ventricular tachyarrhythmias, including ventricular tachycardia and ventricular fibrillation, are life-threatening cardiac conduction disorders that usually occur in the setting of structural heart disease, including coronary artery disease and cardiomyopathies. The management of ventricular tachyarrhythmias begins with the categorization of the arrhythmia to delineate the etiology. Non-sustained ventricular tachycardia is defined as three or more consecutive ventricular complexes at a rate of more than 100 beats per minute with a duration of less than 30 seconds. Sustained ventricular tachycardia occurs when the arrhythmia is longer than 30 seconds. The morphology of ventricular tachycardia is further categorized into monomorphic ventricular tachycardia (single stable QRS morphology) and polymorphic ventricular tachycardia (changing QRS morphologies) [1].

We present a unique case where a patient with exercise-induced ventricular tachycardia was found to have significant coronary artery disease. This is noteworthy because despite the finding of significant coronary artery disease, our patient developed a recurrence of ventricular tachycardia and upon further investigation, the patient was found to have concurrent idiopathic ventricular tachycardia with targetable lesions on the electrophysiologic study.

This article was previously presented as a conference abstract at the American College of Cardiology Together With the World Congress of Cardiology (ACC Together With WCC or ACC.23/WCC) annual scientific meeting in New Orleans, Louisiana, on March 6, 2023.

## Case presentation

A 57-year-old male with a past medical history of hypertension on losartan-hydrochlorothiazide 100-25 mg once daily was initially seen in a clinic for chest discomfort, shortness of breath, and palpitation. The patient denied any other complaints, including dizziness or syncope. Further evaluation was done with an outpatient exercise stress echocardiogram. During the exercise stress test, the patient developed an episode of non-sustained ventricular tachycardia, which lasted for ~25 seconds. The patient was transferred to the emergency room (ER) for further evaluation and management.

**Figure 1:**
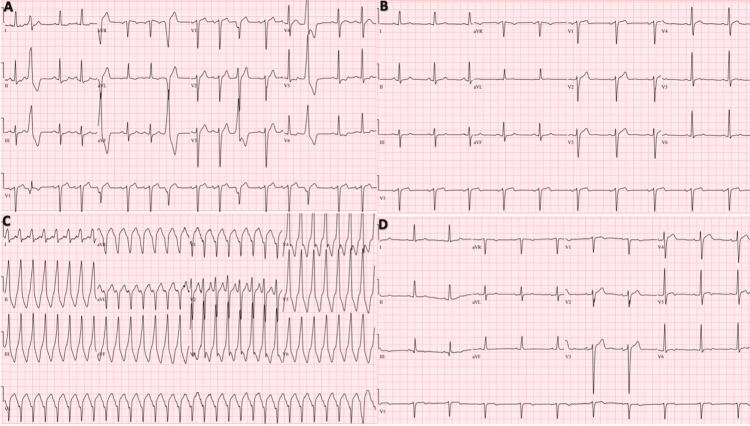
Evolution of the patient’s electrocardiogram (ECG) throughout hospital admission A: ECG on admission showing multiple premature ventricular complexes. B: Post-cardiac catheterization ECG showing normal sinus rhythm (NSR). C: ECG showing sustained, monomorphic ventricular tachycardia. D: Successful radiofrequency ablation to NSR.

In the ER, initial vitals and physical examination were unremarkable. The initial electrocardiogram (ECG) showed normal sinus rhythm with premature ventricular complexes (PVCs) (Figure 1A). Laboratory tests result including troponin of 0.10 ng/mL (reference range: <0.15 ng/mL), B-type natriuretic peptide of 78 pg/mL (reference range: <100 pg/mL), and electrolytes were within normal limits.

The differential diagnosis for exercise-induced non-sustained ventricular tachycardia in our patient included it being a benign non-pathologic finding, coronary artery disease, hypertrophic cardiomyopathy, or idiopathic ventricular tachycardia with an underlying electrophysiologic conduction abnormality.

The next day, the patient underwent cardiac catheterization as part of standard ischemia evaluation of ventricular tachycardia, which revealed significant coronary artery disease requiring intervention. Coronary arteries on cardiac catheterization showed right dominant coronary circulation, 50-60% obstruction of the mid-left anterior descending artery, 40% obstruction of the distal-left anterior descending, 60% obstruction of the first diagonal, 70-80% obstruction of the ramus intermedius, 30% obstruction the proximal- and mid-right of coronary artery, 50-60% obstruction of the right-posterior descending artery, and 70% obstruction of the right posterolateral segment. A drug-eluting stent was placed in the ramus intermedius lesion. The instantaneous wave-free ratio was negative for the mid-left anterior descending and first diagonal arteries. Planned for staged percutaneous coronary intervention of posterior descending and right posterolateral segment lesions.

Following cardiac catheterization, the patient was started on aspirin 81 mg once daily, clopidogrel 75 mg once daily, rosuvastatin 40 mg once daily, valsartan 40 mg once daily, and metoprolol succinate 50 mg once daily. A transthoracic echocardiogram was performed and left ventricular size was normal with an ejection fraction of 50-55% and trivial mitral and tricuspid regurgitation. The patient’s electrocardiogram showed normal sinus rhythm after cardiac catheterization (Figure 1B).

The next day, he had one episode of hemodynamically stable sustained monomorphic ventricular tachycardia lasting for ~4 minutes, when an amiodarone drip was started. Later that day, he had another episode of hemodynamically stable sustained monomorphic ventricular tachycardia, the amiodarone drip was discontinued, and started on diltiazem infusion. While on the diltiazem drip, had another episode of hemodynamically unstable sustained ventricular tachycardia (Figure 1C), and 120 Joules of shock were required for cardioversion. During these episodes, he only complained of palpitations but denied any chest pain, shortness of breath, lightheadedness, or dizziness. 

Several episodes of non-sustained and sustained ventricular tachycardia prompted an urgent electrophysiological study that showed right and left ventricular outflow tract ventricular tachycardia. There was hemodynamically stable, sustained, monomorphic ventricular tachycardia, which was inducible. This tachycardia degenerated to ventricular fibrillation, which was successfully converted to normal sinus rhythm using 200 and 360 Joules in four attempts. Multiple ectopy was seen with right ventricular outflow tract (RVOT) morphology and mitral annular morphology. The most prevalent and likely clinical premature ventricular complexes were in the poster septal aspect of the RVOT. The earliest local activation was found to be ~ 24 milliseconds before QRS onset. Induced ventricular tachycardia (like clinical ventricular tachycardia) - activation mapping showed late activation in RVOT. Given easy degeneration to ventricular fibrillation (during isoproterenol infusion) decision was made not to reinduce the tachycardia but rather perform pace mapping. The best pace map ~ 90% identical to ventricular tachycardia morphology was found underneath the left aortic cusp. This arrhythmia was successfully ablated. Radiofrequency lesions were applied at the left and right ventricular outflow tracts. The repeat electrocardiogram showed normal sinus rhythm (Figure 1D).

All diagnostic study results are summarized in Table 1.

**Table 1:** Inpatient diagnostic studies performed during hospitalization The left column indicates the day of hospital admission and the associated test. The right column indicates the result of the corresponding test.

Test	Result
Electrocardiogram (hospital day 1)	Normal sinus rhythm (NSR) with premature ventricular complexes (PVCs) (Figure 1A)
Cardiac catheterization (hospital day 2)	Right dominant coronary circulation. Mid-left anterior descending artery: 50-60% obstruction, instantaneous wave-free ratio negative. Distal-left anterior descending: 40% obstruction. First diagonal: 60% obstruction, instantaneous wave-free ratio negative. Ramus intermedius: 70-80% obstruction, a drug-eluting stent was placed. Proximal and mid-right coronary artery: 30% obstruction. Right-posterior descending artery: 50-60% obstruction. Right posterolateral segment: 70% obstruction. Planned for staged percutaneous coronary intervention of posterior descending and posterolateral segment lesions.
Transthoracic echocardiography (hospital day 3)	The left ventricular size was normal with an ejection fraction of 50-55%.
Electrophysiological study (hospital day 4)	There was hemodynamically stable, sustained, monomorphic ventricular tachycardia (VT), which was inducible. This tachycardia degenerated to ventricular fibrillation, which was successfully converted to NSR using 200 and 360 Joules in four attempts. Multiple ectopy was seen with right ventricular outflow tract (RVOT) morphology and mitral annular morphology. The most prevalent and likely clinical PVCs were in the poster septal aspect of the RVOT. Earliest local activation was found to be ~ 24 milliseconds before QRS onset. Induced VT (like clinical VT). Activation mapping showed late activation in RVOT. Given easy degeneration to ventricular fibrillation (during isoproterenol infusion) decision was made not to reinduce the tachycardia but rather perform pace mapping. The best pace map ~90% identical to VT morphology was found underneath the left aortic cusp. This arrhythmia was successfully ablated. Radiofrequency lesions were applied at the left and right ventricular outflow tracts.

For the next 48 hours, the patient was symptom-free and had no further episode of ventricular tachycardia on telemetry. He was safely discharged home with a follow-up appointment scheduled within one week and return precautions. The patient presented to the clinic for follow-up after one week of discharge and denied any symptoms including palpitations, chest pain, shortness of breath, dizziness, or syncope.

## Discussion

Ventricular tachycardia symptoms range from having none to shortness of breath, chest pain, shock, or sudden cardiac death. Following ventricular tachycardia, it is imperative to complete further workup to assess the etiology and prevent further episodes. Patients typically undergo cardiac catheterization with coronary angiography to assess for coronary artery disease as a likely culprit, as well as a cardiac echocardiogram to assess for other structural heart diseases. Cardiac magnetic resonance imaging can be considered in selected patients. The absence of structural heart disease in patients with ventricular tachycardia carries a favorable prognosis and is termed idiopathic ventricular tachycardia. Despite its name, more recent diagnostic methods have uncovered electrophysiologic diagnoses with specific sites of origin. The current literature classifies idiopathic ventricular tachycardia into three categories of syndrome; repetitive monomorphic ventricular tachycardia (also called right ventricular outflow tract ventricular tachycardia), paroxysmal sustained ventricular tachycardia, and idiopathic left ventricular tachycardia. Typical sites of arrhythmogenic origin have been described, which are susceptible to radiofrequency ablation for definitive cure [2,3]. These sites include the right and left ventricular outflow tracts, tricuspid annulus, right and left ventricles, inferoapical septum, and aortic cusps. Electrocardiographic patterns have been described that can predict the site of origin in idiopathic ventricular tachycardia [4].

In our present case, the patient experienced an episode of non-sustained ventricular tachycardia during an outpatient exercise stress test, which is a relatively common problem [1]. As part of the standard investigation, the patient underwent cardiac catheterization, which revealed triple-vessel coronary artery disease. As statistically predicted, it was determined that the culprit for exercise-induced non-sustained ventricular tachycardia was likely due to the significant coronary artery disease. However, further stay in the hospital uncovered recurrent, hemodynamically stable, sustained monomorphic ventricular tachycardia that was later followed by an episode of hemodynamically unstable sustained ventricular tachycardia requiring cardioversion. The patient underwent an electrophysiological study after ventricular tachycardia recurrence and was found to have inducible sustained monomorphic ventricular tachycardia, which degenerated into ventricular fibrillation. Ventricular tachycardia morphologies were isolated and radiofrequency ablation was performed successfully.

Recurrent, life-threatening, ventricular tachycardia in our patient highlights a potential dilemma in the standard-of-care investigation for exercise-induced, non-sustained ventricular tachycardia in whom significant coronary artery disease is found upon initial investigation. An electrophysiological study is typically pursued whereupon no coronary artery disease or other significant structural heart disease is revealed. In our search, there is limited literature that details the presence of an electrophysiologic etiology of ventricular tachycardia in patients who are found to also have significant coronary artery disease and do not have other structural heart diseases. One trial performed an electrophysiological study in patients with coronary artery disease who also had heart failure with a left ventricular ejection fraction of 40% or less, and asymptomatic non-sustained ventricular tachycardia [5]. One other study investigated the presence of ventricular tachycardia in patients with hypertrophic cardiomyopathy [6]. Our patient only has coronary artery disease without heart failure or other structural heart diseases.

The recurrence of in-hospital ventricular tachycardia in our patient following the percutaneous coronary intervention of significant coronary artery disease allowed life-saving treatment that is not as readily available outside of a hospital setting. The identified targetable lesion in the electrophysiological study highlights that one cannot presume coronary artery disease is the only, or primary, cause of incidental non-sustained ventricular tachycardia. Further studies are necessary to uncover the prevalence of electrophysiologic etiologies of ventricular tachycardia in patients found to have exercise-induced non-sustained ventricular tachycardia and who are thereafter found to have significant coronary artery disease.

## Conclusions

We report a rare case of idiopathic ventricular tachycardia that was found in a patient with significant coronary artery disease. Based on our literature search, this appears to be the first documented case. Based on the nature of ventricular tachycardia degenerating to ventricular fibrillation, it would have been fatal if interventions such as ablation of the lesions were not performed in a timely manner. This case highlights the importance of considering additional etiologies of exercise-induced ventricular tachycardia despite the presence of significant coronary artery disease.

## References

[1] Buxton AE, Calkins H, Callans DJ (2006). ACC/AHA/HRS 2006 key data elements and definitions for electrophysiological studies and procedures: a report of the American College of Cardiology/American Heart Association Task Force on Clinical Data Standards (ACC/AHA/HRS Writing Committee to Develop Data Standards on Electrophysiology). Circulation.

[2] Belhassen B, Viskin S (1993). Idiopathic ventricular tachycardia and fibrillation. J Cardiovasc Electrophysiol.

[3] Brooks R, Burgess JH (1988). Idiopathic ventricular tachycardia. A review. Medicine (Baltimore).

[4] Enriquez A, Baranchuk A, Briceno D, Saenz L, Garcia F (2019). How to use the 12-lead ECG to predict the site of origin of idiopathic ventricular arrhythmias. Heart Rhythm.

[5] Buxton AE, Lee KL, DiCarlo L (2000). Electrophysiologic testing to identify patients with coronary artery disease who are at risk for sudden death. Multicenter Unsustained Tachycardia Trial Investigators. N Engl J Med.

[6] Adduci C, Boldini F, Palano F (2020). Prognostic implications of nonsustained ventricular tachycardia morphology in high-risk patients with hypertrophic cardiomyopathy. J Cardiovasc Electrophysiol.

